# The Multifaceted Role of microRNA-10b (miR-10b) in Glioblastoma: From Oncogenic Driver to Therapeutic Target

**DOI:** 10.3390/cells15090784

**Published:** 2026-04-26

**Authors:** Ming Chen, Zdravka Medarova, Lisa R. Rogers, Anna Moore

**Affiliations:** 1 Precision Health Program, Michigan State University, 766 Service Road, East Lansing, MI 48824, USA; chenmi26@msu.edu; 2 Department of Radiology, College of Human Medicine, Michigan State University, 846 Service Road, East Lansing, MI 48824, USA; 3 TransCode Therapeutics, Inc., 400 Trade Center, Suite 5900, Woburn, MA 01801, USA; zdravka.medarova@transcodetherapeutics.com; 4 Department of Surgery, College of Human Medicine, Michigan State University, 4660 S. Hagadorn Rd., East Lansing, MI 48823, USA; 5 Department of Neurosurgery, Henry Ford Health, 2799 W. Grand Blvd., Detroit, MI 48202, USA

**Keywords:** glioblastoma, microRNA-10b, oligonucleotide drug delivery, combination therapy

## Abstract

Glioblastoma (GBM) remains one of the most lethal and treatment-resistant human malignancies, characterized by extreme molecular heterogeneity and a highly immunosuppressive tumor microenvironment (TME). MicroRNAs are a set of small endogenous non-coding RNA molecules which play critical roles in various biological processes including carcinogenesis. Recent evidence identifies microRNA-10b (miR-10b) as a regulator of gliomagenesis, with glioblastoma exhibiting a unique state of “oncogene addiction” to this molecule. This review summarizes current research on the mechanistic roles of miR-10b in GBM tumor progression and immune evasion, evaluates innovative antisense oligonucleotide delivery systems, and explores the clinical potential of combining miR-10b inhibition with standard-of-care treatments.

## 1. The Clinical Challenges in Glioblastoma

### 1.1. Existing Treatment Options for GBM

GBM is the most aggressive and lethal primary brain malignancy in adults and remains a major unmet clinical challenge despite decades of research and attempts to develop new therapeutics [1,2,3]. Standard-of-care treatment include maximal safe surgical resection followed by the Stupp protocol, which combines fractionated radiotherapy with the genotoxic drug temozolomide (TMZ) [4,5]. This multimodal approach only modestly prolongs survival, with the median overall survival of 15–18 months, and the five-year survival rate of less than 5% [2,4,6]. Virtually all patients experience tumor recurrence, underscoring the need for new therapeutic strategies that target the fundamental biological drivers of GBM progression and resistance while achieving durable disease control [7,8].

The failure of existing therapies is rooted in several intrinsic features of GBM biology. One of the most significant challenges is the profound molecular and cellular heterogeneity of the tumor [9,10]. GBM comprises multiple co-existing subclonal populations with distinct genetic, epigenetic, and phenotypic profiles. This heterogeneity enables rapid adaptation to therapeutic pressures and promotes the emergence of resistant clones following treatment [11]. In addition, glioma stem cells (GSCs) represent a highly tumorigenic and therapy-resistant subpopulation that contributes to tumor initiation, maintenance, and recurrence [12]. GSCs exhibit enhanced DNA damage repair capacity, resistance to apoptosis, and the ability to repopulate the tumor after cytotoxic therapy, making them a critical barrier to long-term therapeutic success [13].

The tumor microenvironment (TME) in GBM further complicates treatment [14]. GBM is protected by the blood–brain barrier (BBB), a highly selective physiological interface that restricts the passage of most systemically administered therapeutics into the central nervous system. Even in regions where the BBB is partially disrupted, drug delivery remains heterogeneous and insufficient to reach infiltrative tumor cells that migrate into surrounding brain tissue. Moreover, the GBM microenvironment is known to be immunosuppressive, characterized by the presence of tumor-associated macrophages, regulatory T cells, and immunosuppressive cytokines [15]. This environment inhibits natural anti-tumor immune responses and limits the success of immunotherapeutic approaches. Together, these physical and biological barriers create a disease that is extremely difficult to treat [1,16,17,18,19,20,21].

### 1.2. microRNA-10b as an Emerging Target for GBM

microRNAs (miRNAs) have emerged as critical regulators of gene expression networks that control tumor behavior. miRNAs are small (19–22 nucleotide), endogenous, non-coding RNA molecules that modulate gene expression post-transcriptionally by binding to complementary sequences within the 3′ untranslated regions (UTRs) of target mRNAs [22]. This interaction results in translational repression or mRNA degradation, enabling miRNAs to fine-tune complex gene regulatory networks. Importantly, individual miRNAs are capable of regulating numerous target genes simultaneously, enabling coordinated control of entire signaling networks rather than single gene products. Through this broad regulatory capacity, miRNAs are seen as critical modulators of key cellular functions, including cell proliferation, migration, differentiation, apoptosis, and responses to cellular stress [23]. Dysregulation of miRNA expression has become recognized as a defining feature of many cancers, including GBM, where it promotes tumor progression, invasive behavior, and resistance to therapy.

The goal of this review is to provide a synthesis of the available experimental and translational studies on miR-10b, which has emerged as a central driver of glioblastoma pathogenesis and one of the regulators of its invasive and immune-evasive phenotype [16,24,25]. Unlike many oncogenic factors that show variable expression across tumor subtypes, miR-10b is consistently and robustly upregulated in virtually all GBM subtypes. Notably, miR-10b expression is nearly undetectable in normal neuroglial cells, creating a clear tumor-specific expression gradient [25,26,27,28]. This unique feature distinguishes miR-10b from many other therapeutic targets and suggests a high degree of selectivity for targeted intervention.

Functionally, miR-10b promotes tumor cell proliferation, invasion, and survival by regulating multiple downstream targets involved in cell cycle control, apoptosis, and extracellular matrix remodeling [28,29]. Its role as a regulator of gene expression makes it particularly attractive for therapeutic targeting, as its inhibition has the potential to simultaneously change multiple oncogenic pathways [30]. Importantly, experimental studies have demonstrated that inhibition of miR-10b induces apoptosis and growth arrest in glioma cells while having minimal effects on normal neural cells [24,25]. This differential sensitivity suggests that miR-10b-targeted therapies may achieve a favorable therapeutic index, selectively eliminating tumor cells while sparing healthy brain tissue.

Taken together, the combination of GBM’s biological complexity, therapeutic resistance, and delivery challenges necessitates innovative approaches that move beyond conventional single-target strategies. Targeting key regulatory axis such as miR-10b offers a promising avenue to overcome limitations of current therapies by simultaneously modulating multiple oncogenic pathways within a tumor-specific context.

## 2. Molecular Foundations of miR-10b Dependency in GBM

The biological significance of miR-10b in GBM is often described as a state of “oncogene addiction,” where the tumor’s viability is strictly dependent on the continuous expression of this single microRNA [25]. Extensive molecular characterization across diverse glioma cell lines, including U251, LN229, and SF188, as well as patient-derived GSCs, has established that miR-10b loss-of-function leads to inhibition of tumor growth [24,26,29]. In contrast, non-CNS cancer cell lines do not exhibit this absolute requirement, highlighting the specialized evolutionary role of miR-10b in brain tumors [25].

### 2.1. Cytoplasmic and Nuclear Regulatory Mechanisms

The oncogenic influence of miR-10b is mediated through a complex regulatory network that spans both the cytoplasm and the nucleus, modulating a broad array of targets involved in the cell cycle, apoptosis, and cellular plasticity [16,22,31].

**Figure 1:**
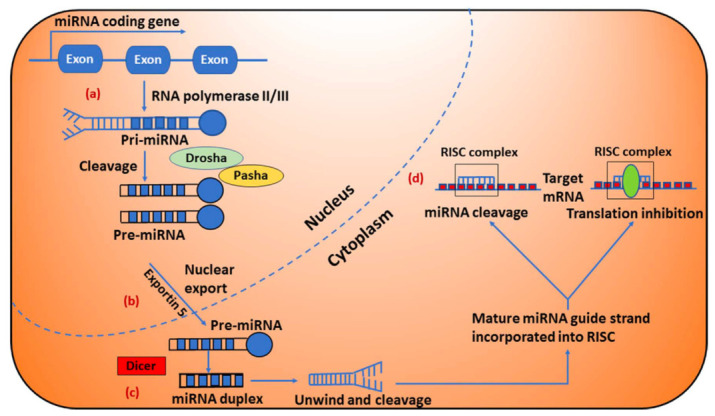
Biogenesis of microRNAs in cells [32]. (a) miRNA genes are typically transcribed by RNA polymerase-II and produce long primary miRNA (pri-miRNA), which are recognized and cleaved in the nucleus by the RNA polymerase III enzyme Drosha. (b) Pri-miRNA is processed to precursor miRNA (pre-miRNA) hairpin like structure in the nucleus by the Drosha/Pasha complex, transported into the cytoplasm by Exportin 5 and further processed by the RNase enzyme Dicer, producing miRNA duplexes. (c) The miRNA duplexes (shown in blue color) are then unwound and the guide strands are selected by Argonaute for integration into RISC. (d) The mature miRNA leads RISC to cleave the mRNA or induce translational repression. Original figure is licensed under a Creative Commons Attribution 3.0 Unported License, https://creativecommons.org/licenses/by/3.0/ (accessed on 22 April 2026).

In the cytoplasm, miR-10b follows the classical microRNA biogenesis pathway, beginning with its transcription by RNA polymerase-II and producing long primary miRNA (pri-miRNA), which are recognized and cleaved in the nucleus by the RNA polymerase III enzyme Drosha (Figure 1a). Next pri-miRNA is processed to precursor miRNA (pre-miRNA) hairpin like structure in the nucleus by the Drosha/Pasha complex, are then transported into the cytoplasm by Exportin 5 (Figure 1b) [22,32]. Further processing by the RNase III enzyme Dicer produces mature miRNA duplexes (Figure 1c). The miRNA duplexes are then unwound and the guide strands are integrated by Argonaute into the RNA induced silencing complex (RISC). The mature miRNA then leads RISC to cleave the mRNA or induce translational repression (Figure 1d) [22,31].

The cytoplasmic targets of miR-10b include key tumor suppressors such as HOXD10, PTEN, CDKN1A/p21, CDKN2A/p16, and pro-apoptotic factors like BCL2L11/Bim and Apaf-1 [16,28,33,34,35,36,37,38]. By suppressing these genes, miR-10b effectively bypasses cell cycle checkpoints and evades programmed cell death, facilitating rapid proliferation and survival [16,29,33,39]. Furthermore, miR-10b is a potent inducer of the epithelial–mesenchymal transition (EMT) in glioma cells [33,40]. The inhibition of E-cadherin by miR-10b promotes a more mobile and invasive cellular phenotype, allowing tumor cells to infiltrate the surrounding brain parenchyma which is a primary cause of local recurrence [33].

Recent discoveries have expanded functional repertoire of miR-10b to include a unique nuclear role [16,41]. Specifically, studies have demonstrated that miR-10b binds to the core spliceosomal small nuclear RNA (snRNA) U6 in various cancer cells, but this intermolecular interaction is prevalent in glioma cells [16,41]. This interaction disrupts the critical function of U6 in the spliceosome by reducing its level and stability at the same time, leading to widespread alterations in the splicing patterns of numerous genes implicated in gliomagenesis [16,41]. The authors suggest the mechanistic relationships between miR-10b binding to U6 and its effects on U6 modifications, conformation, and destabilization. They also propose that miR-10b binding leads to a U6 structural-conformational reorganization that, in turn, alters protein binding of some snRNP proteins (e.g., SART3, PRPF8) and enzymes catalyzing U6 modification, including writers and erasers (e.g., methyltransferase METTL16 and pseudouridine synthase Pus1). This, in turn, regulates U6 modifications, spliceosome assembly, and splicing reactions. In such a scenario, the lowered levels of U6 could be a consequence of the reduced U6 incorporation in the snRNPs and miR-10b-mediated effects on U6 secondary structure, which is tightly controlled during the splicing cycle, must play a critical role in the downstream effects on mRNA splicing. This dual-compartment activity underscores the multi-modal oncogenic nature of miR-10b, as it simultaneously silences tumor suppressors at the translational level while modulating the fundamental genetic architecture of the cell through splicing interference [16,41].

### 2.2. Genomic and Epigenetic Integration

The dysregulation of miR-10b is frequently linked to broader genomic alterations characteristic of primary GBM, such as epidermal growth factor receptor (EGFR) amplification and loss of heterozygosity on chromosome 10q [42,43]. Specifically, mutations in the tumor suppressor gene located on 10q23.31 activate the PI3K/AKT signaling pathway, which has been shown to be both a driver of miR-10b expression and a downstream target of its regulatory network [42,44]. In this context, miR-10b acts as an essential mediator of pathway crosstalk, reinforcing the aggressive characteristics of GSCs, which are responsible for self-renewal, differentiation, and overall therapy resistance [26,45]. The miR-10b target genes and their roles are summarized in Table 1.

## 3. MiR-10b and the Immunosuppressive GBM Microenvironment

GBM is characterized by an immunologically “cold” environment, defined by the scarcity of effector T cells and the abundance of immunosuppressive factors that hinder the success of standard immunotherapies such as immune checkpoint inhibitors (ICIs) [1,48,49]. MiR-10b plays an important role in TME as it modulates the behavior of both tumor cells and the infiltrating immune population through paracrine and exosomal communication [17,33,46,50].

### 3.1. Adaptive Immunity: The TET2/PD-L1 Axis

A critical mechanism of immune escape in GBM involves the Programmed Death-1 (PD-1)/Programmed Death-Ligand 1 (PD-L1) checkpoint pathway [46]. Research has elucidated a novel regulatory axis where miR-10b-5p impairs the Ten-eleven translocation 2 (TET2)-mediated inhibition of PD-L1 transcription [46,51]. Under physiological conditions, TET2 protein acts as a transcriptional repressor by recruiting histone deacetylases HDAC1 and HDAC2 to the promoter region of the PD-L1 gene. In GBM, however, the universal upregulation of miR-10b leads to the direct suppression of TET2 [46].

The resulting TET2 deficiency allows for the robust expression of PD-L1 on the tumor cell surface, which, upon binding to the PD-1 receptor on cytotoxic T lymphocytes, induces T-cell apoptosis and functional exhaustion [46]. Assays involving the co-culture of miR-10b-inhibited U251 cells with T cells have shown significant reductions in T-cell apoptosis and a marked increase in T-cell-mediated cytotoxicity, as measured by lactate dehydrogenase (LDH) release [46]. This finding suggests that miR-10b not only promotes intrinsic tumor growth but also actively disarms the adaptive immune system, making its inhibition a potential strategy for sensitizing GBM to anti-PD-1/PD-L1 therapies.

### 3.2. Innate Immunity: Macrophage Polarization

The GBM environment is heavily infiltrated by tumor-associated macrophages (TAMs), which can constitute up to 50% of the total tumor mass [52,53]. These TAMs exist in a spectrum between the tumor-suppressive M1-like phenotype and the tumor-supportive, immunosuppressive M2-like phenotype [54,55,56]. GBM cells secrete various factors and metabolites, such as lactic acid (LA) produced via hypoxia-induced glycolysis, to drive macrophages toward the M2 state [47,57]. MiR-10b regulates this polarization through exosomal communication [47]. Hypoxia-stimulated glioma cells release extracellular vesicles (EVs) enriched with miR-10b-5p [17,47]. Once internalized by macrophages, miR-10b targets the E3 ubiquitin ligase NEDD4L, which under normal conditions promotes the ubiquitination and degradation of PIK3C [47]. The depletion of NEDD4L results in the stabilization of PIK3CA and the subsequent activation of the PI3K/AKT signaling pathway, which is a required step for M2 macrophage polarization [47]. This polarization is further supported by other signaling factors such as the Oncostatin M receptor (OSMR), which activates the JAK/STAT3 pathway to enhance the M2 fraction within the TME [54].

### 3.3. Recruitment of Suppressor Cells

The influence of miR-10b extends to the active recruitment of myeloid-derived suppressor cells (MDSCs) and regulatory T cells (Tregs), which create a beneficial environment for tumor progression [31,58,59]. GBM cells produce specific chemokines, such as CCL2 and CCL22, that preferentially recruit CCR4-expressing Tregs into the tumor parenchyma [59,60]. The stabilization of this suppressive environment is often driven by TGF-β, which is not only an inducer of miR-10b but is also secreted by miR-10b-driven M2 macrophages, creating a self-sustaining loop of immunosuppression and growth [33,61,62].

Furthermore, the “MDSC-miRs”, a cluster including miR-10b, miR-155, and miR-146a, are frequently found in GBM-derived EVs and have been identified as predictors of resistance to immunotherapy [58]. These miRs inhibit the maturation of myeloid cells and promote their skewing toward the MDSC phenotype, which further impairs cytotoxic CD8+ T cell and natural killer (NK) cell responses [49,58,63].

The immune cell roles and changes in biological activity by miR-10b inhibition in GBM TME are listed in Table 2.

## 4. Antisense Oligonucleotide Design and Innovative Delivery Systems for miRNA Therapeutics

Nucleic acid-based therapies including miRNA therapies offer innovative solution for diseases including cancer, cardiovascular, neurodegenerative, metabolic, infectious disease, and orphan genetic syndromes, which remain major therapeutic challenges (reviewed in [64]). Major advantages include versatility in reaching previously “undruggable” pharmacological targets in a broad spectrum of diseases, relative safety, efficacy and tolerability. For miRNA-based intervention the most efficient, and consequently especially attractive feature, is the broad spectrum of targets that can be regulated by a single miRNA [65]. However, despite these advantages, application of antisense oligonucleotides (ASOs)-based therapeutics into clinic has been slow due to unresolved challenges in delivery, RNA stability, immunogenicity, and large-scale production [64].

### 4.1. Considerations for the ASO Design

ASOs bind with high affinity to the target miRNA within the RNA-induced silencing complex (RISC). This binding prevents the miRNA from interacting with its mRNA targets resulting in derepression of the target gene (in case of miR-10b, the target is *HOXD10* gene [66]). Guiding principles for ASO design are thoroughly reviewed in [67]. Here, we summarize the most important considerations that drive the design of therapeutics that are either in clinic or in clinical trials. Anti-miR oligonucleotides-based mechanism of action relies on the complementary base pairing of the oligonucleotide sequence to its target miRNA. For that reason, 21–23 nucleotides long ASOs are typically designed to provide a perfect complementarity to the target, especially in the seed region [68]. To enhance stability and target affinity, ASOs can be chemically modified. For example, 2′-O-methylation (2′-OMe) modification of all ribose moieties in the oligonucleotide chain increases metabolic stability and binding affinity [69]. Further, the use of locked nucleic acids (LNAs) that features a “locked” ribose ring connected by a methylene bridge between the 2′-O and 4″-C atoms provides an enhanced thermal stability and binding affinity [70]. Phosphorothioate (PS) linkage, where the non-bridging phosphate oxygen is substituted by a sulfur in the phosphodiester bond, usually at the 5′ and 3′ ends, protects the oligo against exonuclease degradation [71].

Delivering oligonucleotides to the Central Nervous System (CNS), and specifically to GBM, represents a challenge due to the presence of the BBB. Optimization usually involves balancing size, charge, and lipophilicity. For typical unmodified ASOs with molecular wight around 5000 Da passive diffusion across BBB is hindered, requiring the application of delivery vehicles (described in Section 4.2). Negative charge of the phosphorothioate linkage limits cell membrane permeability, also requiring cellular transport arrangement. Since the PS backbone also increases circulation time by binding to serum proteins, particularly albumin [67], thus increasing the size of the complex, special considerations are required to ensure efficient renal clearance while maintaining efficient delivery to tumor cells. Finally, the addition of a 3′-cholesterol group in some designs is used to increase the lipophilicity and improve tissue penetration and cellular uptake of the ASO [72].

While over a dozen antisense oligonucleotides (ASOs) have been FDA-approved for various diseases [73], none has been approved for cancer therapy. As mentioned above, GBM is an especially difficult-to-treat cancer due to the challenges in the effective delivery of RNA inhibitors or gene-editing tools to the diffuse, infiltrative tumors in the brain [16,39,74]. In the context of miR-10b-targeted therapy, to overcome the BBB and ensure specific uptake by tumor cells, researchers have developed two primary nonviral platforms: magnetic nanoparticles (MN) and lipid nanoparticles (LNP) [16,39].

### 4.2. Magnetic Nanoparticle-Antagomir Delivery (MN-anti-miR10b)

The MN-anti-miR10b platform utilizes a magnetic iron oxide core conjugated to antisense oligonucleotides (antagomirs) specifically designed to sequester mature miR-10b molecules [39]. This strategy offers several unique advantages:

1. Protection from Degradation by Nucleases: The nanoparticles are coated with the dextran polymer which protects the short 15-mer ASO from enzymatic degradation most likely by creating a steric hindrance to large nuclease molecules and restricting access to the active center of the enzyme.
2. Circulation half-life: Nanoparticles are significantly larger than non-conjugated antisense oligos, and provide therapeutic benefits by increasing its circulation half-life [39].
3. MRI Guidance: The magnetic core serves as an imaging reporter, allowing for the real-time monitoring of delivery and accumulation within the tumor core and invasive margins using T2-weighted magnetic resonance imaging [24,39].
4. Increased BBB Penetration: Unlike unconjugated oligos, magnetic nanoparticles are designed for systemic administration and have demonstrated the ability to cross the BBB and reach orthotopic tumor sites in humanized murine models [24,39]. Therapeutic studies in orthotopic GBM models have shown that intravenous administration of MN-anti-miR10b leads to a statistically significant extension of median survival (54.4 days vs. 44 days in control groups) and a 3.5-fold increase in intratumoral apoptotic activity [39]. This platform forms the foundation for the clinically tested therapeutic TTX-MC138, which is currently undergoing evaluation in humans [39].
5. Multiple studies with magnetic nanoparticles used as the antisense oligo carriers confirmed their safety in small [75,76,77] and large animals [78].

### 4.3. Lipid Nanoparticle-Mediated CRISPR-Cas9 (miRTEN)

A more permanent therapeutic intervention involves the ablation of the miR-10b gene itself using the CRISPR-Cas9 system [16,25]. Researchers developed “miRTEN,” a lipid nanoparticle (LNP) formulation encapsulating Cas9 mRNA and a miR-10b-targeting single-guide RNA (sgRNA) [16]. This system leverages the scalability and safety of LNPs, which have already been validated for mass production and clinical use in other indications [16].

Intracerebroventricular (ICV) injection of miRTEN used in this study demonstrated several critical therapeutic effects:

1. Widespread Editing: ICV administration enables the distribution of the gene-editing machinery across the entire brain, targeting both the primary tumor and the distant invasive cells that are often missed by surgical resection [16,74].
2. Bystander Effect: Interestingly, miRTEN-edited cells were shown to produce a secretome that reduced the growth and viability of neighboring, non-edited glioma cells [16,79]. Conditioned media from edited GSCs significantly inhibited spheroid growth in naive GBM cell lines (GBM8 and GBM62), suggesting that high-efficiency editing of 100% of the tumor mass may not be required for clinical success [16]. PGK1 and IGFBP2 were identified as the primary secreted factors responsible for inducing this selective cell death. Crucially, this treatment targeted malignant cells while leaving normal neuroglial cells unharmed. These findings suggest that gene editing therapies could be successful in treating heterogeneous tumors even without reaching every single cell [79].
3. Immune Memory: In immunocompetent models, miRTEN-treated mice that survived the initial challenge rejected the secondary challenge of new tumor cells (CT2A), demonstrating the development of a durable, tumor-specific immune memory [16]. This effect correlated with a marked increase in the infiltration of cytotoxic T cells and the upregulation of activation markers such as TNFα, IFNγ, and Granzyme B [16].
4. Limitations of CRISPR/Cas9 gene editing: Critical limitations and risks associated with CRISPR/Cas9 include genomic instability, delivery challenges, and long-term safety concerns. A major technical limitation of canonical CRISPR/Cas9 is its reliance on creating double-strand breaks (DSBs) in DNA [80] which can result in large-scale rearrangements, retrotransposition, and whole chromosome loss [80,81]. In addition, CRISPR induces permanent and irreversible genomic changes, raising concerns about unintended off-target effects and long-term consequences in healthy tissues that may be inadvertently transfected. Clinical effectiveness is further limited by delivery challenges, as CRISPR/Cas9 systems are large and complex, reducing in vivo diffusion and efficiency. In brain tissues, therapeutic distribution may extend only a few millimeters from the intracerebroventricular (ICV) injection site, limiting treatment of deep-seated disease [82]. Moreover, incomplete targeting of all cells can allow therapy-escaping cells to drive tumor recurrence [79]. When targeting elements such as miRNAs, single-guide RNA approaches may be less robust than strategies using two guide RNAs to remove the entire precursor structure [16]. Finally, off-target editing at unintended genomic sites remains a persistent risk even when on-target efficiency is high [80,81].

The comparison of these two non-viral delivery platforms for miR-10b targeting is summarized in Table 3.

## 5. Therapeutic Synergies and Clinical Translation

Given the inherent complexity and heterogeneity of GBM, a monotherapeutic approach is unlikely to provide a definitive cure [1,83]. The integration of miR-10b targeting into multimodal strategies offers the potential for profound synergistic effects, particularly when paired with chemotherapy or advanced immunotherapies [16,83,84].

### 5.1. Combination with Chemotherapy (TMZ)

The standard treatment with TMZ often leads to the development of chemoresistance, in part by paradoxically increasing the expression of miR-10b [29,39]. Preclinical studies using human GBM cell lines (U251 and LN229) have demonstrated that sequence-dependent combination therapy where miR-10b inhibition precedes TMZ treatment significantly enhances apoptosis and reduces cell migration compared to either treatment alone [39]. This strategy effectively lowers the therapeutic threshold for TMZ, potentially allowing for reduced doses that minimize systemic side effects while maintaining anti-tumor efficacy [16,39].

### 5.2. Immunotherapy Integration

Adoptive cellular therapies, such as Chimeric Antigen Receptor (CAR) T-cell therapy, have faced significant challenges in GBM due to T-cell exhaustion and the “cold” immune environment [1,42,83]. However, the ability of miR-10b inhibition to increase cytotoxic CD8+ T cell infiltration and reduce immunosuppressive factors (such as IL-4, IL-10, and TGF_β_) provides a rational basis for this combination [16]. Specific “fourth-generation-like” designs are being explored where CAR constructs also incorporate therapeutic miRNAs or precursors [85]. For example, CAR-T cells targeting IL-13R2 or EGFRvIII could be co-administered with miR-10b inhibitors to create a more permissive environment for T-cell persistence and proliferation [1,85]. Similarly, miR-10b targeting could enhance the efficacy of peptide-based or dendritic cell (DC) vaccines [84,85]. While vaccines aim to induce a robust anti-tumor immune response, their success is often curtailed by the local immunosuppressive milieu [84,85]. By neutralizing miR-10b, clinicians can effectively remove the “molecular shield” of the tumor, allowing vaccine-induced cytotoxic T lymphocytes to more effectively recognize and eradicate the malignancy [16,46,85].

### 5.3. Multi-Targeted RNAi Strategies

The evolutionary plasticity of GBM allows it to adapt to the inhibition of single pathways [45,86]. Systematic reviews of preclinical studies indicate that combinational RNA interference (RNAi) therapies which target multiple oncogenic miRNAs or pathways simultaneously can reduce tumor volumes to 22.85% of original size, compared to only 54.75% for single RNAi therapies [86]. Various mechanisms can contribute to this effect including therapeutic strategies to upregulate downregulated pro-apoptotic proteins such as BAX, BAK and BH3-only proteins to restore apoptotic signaling [87]. Another example of multifaceted strategies is targeting angiogenesis together with pro-metastatic factors such as EMT [88,89]. Targeting miRNA clusters involved in cell cycle regulation and apoptosis has also been investigated. For example, miRNA 106b∼25 clusters comprising miRNA 106b, miRNA-93, and miRNA-25 are targeted using respective anti-miRNAs and have significantly inhibited proliferation, invasion, and migration while promoting apoptosis [90]. These studies showed efficacy for gastric cancer but can also be investigated in the context of GBM. For example, pre-clinical studies in GBM showed potential of targeting miR-21 implicated in suppressing tumor suppressors [91]. Therefore, combining the inhibition of miR-10b with other regulators like miR-21, or with the restoration of tumor-suppressor miRs like miR-7 or miR-34a, represents the next logical step in developing comprehensive RNA-based GBM treatments [22,42,61,86].

### 5.4. Clinical Translation and Human Clinical Trial Updates

The translation of miR-10b-targeted therapies from preclinical success to clinical utility is advancing rapidly, primarily through the efforts of biotechnology innovators focusing on systemic RNA delivery. Below we discuss several innovations that are close to clinic or already in clinical trials.

RGLS5579:

RGLS5579 is a novel oligonucleotide designed to inhibit miR-10b [92,93]. Although clinical trials of RGLS5579 have not yet been initiated, RGLS5579 increased median survival as a monotherapy, and markedly extended median survival time in combination with TMZ in an orthotopic mouse model in which human GMB tumor cells were implanted into the brains of immunocompromised mice.

TTX-MC138:

TTX-MC138 represents the first-in-class miR-10b inhibitor to enter human clinical trials [92]. This therapeutic candidate targets the regulator of metastatic cell viability in multiple indications, including breast, pancreatic, and colorectal cancers, as well as glioblastoma [92,93,94]. In 2024 and 2025, several key milestones related to safety and preliminary efficacy were reached. Preliminary data from a Phase 0 clinical trial using radiolabeled TTX-MC138 confirmed that even a microdose was well-tolerated and resulted in significant inhibition of miR-10b in the blood. The increase in radioactive lesion-to-blood ratios suggested active uptake by cancerous tissues. By October 2025, the Phase 1a portion of the trial evaluating safety and tolerability across escalating dose levels met its primary endpoint. No significant safety concerns or dose-limiting toxicities were reported in the 16 treated patients, several of whom maintained stable disease without evidence of progression. Following the positive readout from Phase 1a, a Phase 2a clinical trial is anticipated. A limitation of these trials is that only patients with non-GBM solid tumors were recruited. To include GBM patients, it is necessary to conduct imaging studies demonstrating accumulation of TTX-MC138 in tumors, along with confirmation of target engagement. Furthermore, combination studies with standard-of-care therapeutics in animal models including PDX models are needed prior to embarking on trials in patients. Encouragingly, recent preclinical publications have demonstrated that TTX-MC138 is effectively delivered to human GBM tumors implanted in murine brains after intravenous injection, resulting in a five-fold increase in tumor cell death and significant survival benefits [24,39]. These findings strongly support the expansion of TTX-MC138 trials into the neuro-oncology space.

### 5.5. Diagnostic and Prognostic Biomarker Potential

Beyond its role as a therapeutic target, miR-10b serves as a highly specific diagnostic tool. A clinical study (NCT01849952) has identified miR-10b as a prognostic and diagnostic marker in high-grade gliomas. By examining tumor, blood, and cerebrospinal fluid (CSF) samples obtained from patients over a period of two years, investigators aim to correlate miR-10b expression patterns with tumor grade, genotypic variations, and patient survival.

MiRs in the CSF have proven particularly useful for identifying GBM activity and distinguishing it from other metastatic brain cancers [95]. Because more than 95% of the miRNAs found in CSF extracellular vesicles are also present in crude CSF, this provides a minimally invasive liquid biopsy method to monitor disease progression and response to anti-miR-10b therapy in real-time [50,95].

## 6. Conclusions and Future Outlook

MiR-10b represents a unique biomarker in GBM. Its essential role in tumor survival and immune evasion, combined with the lack of expression in normal brain tissue, creates a significant therapeutic opportunity. While challenges in delivery and intratumoral heterogeneity remain, the advent of LNP-mediated gene editing and MRI-guided nanotherapies offers a promising path toward converting this universally lethal disease into a manageable condition.

The current transition of miR-10b inhibitors into human trials, specifically the success of the TTX-MC138 safety assessments, marks the beginning of a new era in RNA-based neuro-oncology. As research continues to unravel the intricate role of miR-10b in immune evasion particularly through the TET2/PD-L1 axis and M2 macrophage polarization, it becomes increasingly clear that miR-10b inhibition is not merely a growth-suppressive strategy, but a potent immunomodulatory intervention.

The ultimate goal for GBM therapy will likely involve a personalized, multi-targeted approach where miR-10b inhibition serves as the cornerstone for warming up the tumor microenvironment. By integrating this targeted intervention with the standard-of-care and next-generation immunotherapies, clinicians may finally be able to resolve the formidable defenses of GBM and provide a meaningful extension of life for patients facing this devastating diagnosis.

## Figures and Tables

**Table 1:** Summary of miR-10b Target Genes and Biological Consequences.

Target Gene/Complex	Regulatory Compartment	Primary Biological Role	Consequence of miR-10b Inhibition	References
CDKN1A/p21	Cytoplasm	Cell cycle inhibition (G1/S)	Restoration of cell cycle control	[16]
BCL2L11/Bim	Cytoplasm	Initiation of apoptosis	Induction of programmed cell death	[16]
PTEN	Cytoplasm	PI3K pathway antagonism	Suppression of survival signaling	[33,37,38,42]
Apaf-1	Cytoplasm	Apoptosome assembly	Enhanced apoptotic sensitivity	[33]
E-cadherin	Cytoplasm	Cellular adhesion	Reduced invasive potential	[33]
U6-snRNA	Nucleus	mRNA splicing catalysis	Restoration of normal splicing patterns	[16,41]
TET2	Cytoplasm	Epigenetic DNA demethylation	Decreased PD-L1 transcription	[46]
NEDD4L	Cytoplasm	Ubiquitin ligase activity	Inhibition of PI3K/AKT via PIK3CA	[47]
HOXD10	Cytoplasm	Acts as a tumor suppressor by preventing cell migration, invasion and blood vessel formation (angiogenesis)	inhibit GBM growth and invasion	[28,34]

**Table 2:** Immune Cell Modulation in the GBM TME.

Cell Type	Phenotype	Role in GBM	Modulation by miR-10b Inhibition	References
CD8+ T Cells	Effector	Direct tumor cell killing	Increased infiltration and activation (IFNγ, Granzyme B)	[16]
TAMs	M2-like	Immunosuppression and invasion	Shift toward M1-like phenotype; reduced immunosuppressive factor release	[46,47]
MDSCs	Suppressor	T-cell inhibition and metastasis	Reduced recruitment; impaired suppressive capacity	[58,61]
Tregs	Suppressor	Evasion of immune surveillance	Decreased recruitment (via TGFβ/CCL22 modulation)	[31,59]
GSCs	Stem-like	Recurrence and drug resistance	Loss of viability; “addiction” to miR-10b is lethal	[25]

**Table 3:** Comparison of Non-Viral Delivery Platforms for miR-10b Targeting.

Feature	Magnetic Nanoparticles (MN)	Lipid Nanoparticles (LNP)
Payload	Antisense Oligonucleotides (ASO)	Cas9 mRNA + sgRNA
Mechanism	Transient Sequestration of miRNA	Permanent Gene Ablation
Delivery Route	Systemic (IV)	Local (ICV)
Imaging	MRI Reporter (clinical)	Fluorescent Reporters (non-clinical)
Primary Advantage	Real-time monitoring of delivery	Induces durable immune memory
Current Stage	Phase 1/2 Clinical Trials (TTX-MC138; NCT06260774)	Preclinical Investigation

## Data Availability

No new data were created in this review.
